# Pre-exposure prophylaxis in Southern Africa: feasible or not?

**DOI:** 10.7448/IAS.18.4.19979

**Published:** 2015-07-20

**Authors:** Willem Daniel François Venter, Frances Cowan, Vivian Black, Kevin Rebe, Linda-Gail Bekker

**Affiliations:** 1 Wits Reproductive Health and HIV Institute (Wits RHI), University of Witwatersrand, Johannesburg, South Africa; 2 Research Department of Infection and Population Health, University College London, London, UK; 3 Health4Men, Anova Health Institute, Cape Town, South Africa; 4 IIDMM, UCT Health Science Faculty, Cape Town, South Africa

**Keywords:** PrEP, HIV, AIDS, South Africa

## Abstract

**Introduction:**

Southern and Eastern Africa bear the brunt of the AIDS epidemic, and current prevention interventions remain inadequate. Antiretroviral-based pre-exposure prophylaxis (PrEP) is gaining momentum as an effective prevention intervention.

**Discussion:**

Discussions have been started on how this strategy could be employed in Africa such that the populations most in need can be reached urgently for the greatest impact. This requires the selection of specific risk groups and service environments in which PrEP can be distributed safely and cost effectively while being mindful of any ethical issues.

**Conclusions:**

Given the need for an integrated public health approach to this, a number of potential populations and opportunities for PrEP distribution exist and are discussed in this commentary.

## Introduction

Southern and Eastern Africa bear the brunt of the AIDS epidemic. Three out of every four people in the world commenced on antiviral therapy live in this region. South Africa alone has a remarkable 2.5 million individuals on treatment in 2014 – the largest HIV-positive population by number in the world [1].

The treatment pool in the region is ever expanding. The epidemic is firmly established within the generalized heterosexual community, but local key populations with extraordinarily high incidence, including sex workers (SWs) and men who have sex with men (MSM), as well as adolescent girls and young women make focused HIV prevention strategies that include antiretroviral pre-exposure prophylaxis (PrEP) seriously worth considering [2]. Furthermore, it may well be possible that HIV transmission attributable to particular populations in Africa, for example, fishing populations in Uganda, has been under-estimated [3, 4].

### PrEP: the evidence for effectiveness

PrEP administered to HIV-negative individuals has been shown in a number of randomized controlled trials to be effective at preventing HIV acquisition. In discordant heterosexual couples, in which adherence to the daily combination antiretroviral therapy (ART) (tenofovir (TDF)/emtricitabine (FTC) or Truvada) was particularly good, PrEP reduced HIV acquisition by 75% [5]. While the reduction in HIV acquisition among MSM in the Global iPrEx study was less striking, effectiveness was shown overall and improved relative to the number of pills taken every week and was highest (92%) among those with detectable blood levels [6]. Benefit was greatest among those men who were most likely to have unprotected receptive anal intercourse [6]. This finding has been borne out in the open label study that followed, and mounting evidence to confirm these findings is being derived from further studies and demonstration projects in MSM around the world [7–9].

Unfortunately, effectiveness evidence in young heterosexual women, to date, is mixed. Relatively good efficacy was shown among young women in two African clinical trials, but two others conducted in the region: FEM-PrEP and VOICE failed to show any efficacy of PrEP in women, due to low product use [4, 10, 11]. Research to understand the reasons for poor adherence in the context of placebo controlled trials found that the unknown efficacy, challenges with taking a daily treatment given the social risks and lack of support from partners appeared to discourage adequate product use [12]. It is likely that adherence will be greater among individuals who choose to take open label PrEP having made their own risk benefit assessment. Recent data from Cape Town confirmed that when women were given PrEP in an open label fashion in three dosing strategies including daily, intermittent with a post sex act boost and pericoital dosing, the majority of women took PrEP, although those in the daily arm were more adherent and more sexual acts were “covered” by PrEP usage [13]. While we have some modelling evidence that less adherence (as few as 4 pills per week) in MSM still can be effective in reducing infections presumably because of high concentration of drug in rectal mucosae, we do not have similar evidence in heterosexual women [14].

Both tenofovir (TDF) and emtricitabine (FTC) and the combination agent are extensively used in Africa, are available in generic formulations, and form part of the WHO's recommended first-line HIV treatment [15]. Although the US Government's Food and Drug Administration (FDA) has approved both tenofovir and Truvada (the originator combination of tenofovir and emtricitabine) for a prevention indication in both men and high-risk women in 2012, these agents are not yet licensed for prevention anywhere in Africa. The Medicines Control Council of South Africa is currently reviewing licensure in South Africa for this indication.

Relevant to the heterosexual epidemic in Africa are the novel methods for delivering PrEP that are being explored alongside the research to determine effectiveness of oral and topical agents. For example, trials testing the effectiveness of using a vaginal ring to deliver antiretrovirals are ongoing: a dapivirine-impregnated ring is being evaluated, with results due in 2016. This monthly vaginal ring could overcome the need for daily or coitally dependent adherence and potentially, as a multi-purpose technology (combining contraceptive as well as antiviral activities) may be particularly suited to the needs of adolescent women [16]. Additionally long-acting injectable depot antiretroviral agents for prevention that also have the possibility of being combined with contraceptive agents are starting safety phase trials [17].

While topical PrEP in the form of coitally administered pre- and post-vaginal dosing of a 1% tenofovir gel microbicide has been shown to be partially effective in preventing both HIV and HSV infections in young heterosexual women in a single trial in South Africa [18], a second, similar but larger, multi-site trial did not show efficacy [19]. A formulation of 1% tenofovir gel suitable for rectal mucosa is being developed for MSM and anal sex use and is currently in early clinical trials.

While clinical and efficacy data are accruing on tenofovir and tenofovir/emtricitabine PrEP usage, there have also been a number of publications over the last few years exploring the impact and cost effectiveness of this strategy in the region and in South Africa in particular. A recent systematic review of PrEP models noted that the effect of promoting PrEP to high-risk groups is highly dependent on sexual mixing patterns in the population and levels of heterogeneity in HIV risk [20]. Most of the models reported show remarkable impact in terms of infections averted, lives saved and reductions in HIV incidence, and this has been shown in both high-incidence and lower-incidence settings [21–26]. Modelling studies underscore that population-level coverage and effectiveness (which is dependent on adherence) are the main determinants of the number of infections averted and that implementation of a combination of ART and PrEP prevents more infections in a population than a programme that delivers exclusively either ART or PrEP alone. Other modelling studies suggest that a high “background” level of ART coverage is likely to increase the cost per HIV infection prevented by PrEP [25, 26].

Recent costing models have also shown that depending on product cost and coverage, the intervention of either systemic or topical PrEP is cost effective in South Africa especially if deployed to populations with particularly high incidence [21–24]. Under optimistic conditions, Walensky showed that PrEP could be cost saving in the South African context [23]. Verquet and colleagues concluded from their study that PrEP would have maximum impact and be cost effective in Southern Africa especially if male circumcision rates were low [26]. They also concluded that PrEP was best utilized as a targeted intervention added to existing strategies for epidemic control. Pretorius and colleagues examined PrEP benefit next to treatment scale–up, and they described a window of opportunity where PrEP would be most cost effective before ART has been adequately scaled up to exert prevention impact [25].

Since oral PrEP with a tenofovir-based regimen is the only PrEP currently available, the rest of this commentary focuses on this intervention.

## Discussion

### PrEP in southern/eastern Africa: feasibility

PrEP as a public health intervention is not insignificant, especially if targeted at specific higher incidence or key populations. The individuals from such populations must be found, without added stigmatization or labelling, and then be voluntarily linked to prevention programmes in which PrEP is offered. In addition, current guidelines and PrEP medications require toxicity screening that adds complexity to large-scale primary care PrEP distribution. Finally, the counselling and frequent repeated HIV testing required by current guidelines pose further challenges to full-scale implementation [27, 28]. Clinics are busy, often impersonal, and rarely offer routine screening for any intervention unless located in verticalized services. That said, antiretroviral treatment and prevention of mother to child transmission (PMTCT) have been relatively well integrated in many primary health care settings, using simplified algorithms based on the WHO public health approach. Simplification of PrEP administration, reassurance around issues of safety and relaxation of monitoring requirements will facilitate PrEP distribution by nurses supported by community care workers at primary care level.

In some countries in the region antiretroviral provision for patients well-established on treatment is moving out of health facilities and into community-based distribution centres where community care workers are instrumental in their smooth running – it is conceivable that PrEP provision would need to follow a similar path given the overcrowding of health facilities [29]. Frequent (three monthly) HIV testing to detect recent seroconversions is a key component of current PrEP programmes and is likely to become a bottleneck at health facilities as well as a disincentive to continuous and safe use of PrEP. Innovative ways of HIV self-testing and alternative models of drug delivery may be necessary. HIV self-testing is a topical issue within the region, and commercial developers have expressed interest in making these available in the region. Integrating reliable self-testing into PrEP programmes would help simplify and potentially reduce cost. South Africa has a well-funded and extensive state sector, an innovative private health sector and an extensive laboratory monitoring system, which means that access to PrEP may indeed be feasible. In other southern and eastern African countries, where the resource constraints are greater, there is still the potential to implement PrEP either through the public health system (as with PMTCT programmes) or supported by non-governmental organizations (as for condom promotion and circumcision programmes). A number of demonstration projects or open label projects are underway or are planned which will help to elucidate some of the implementation issues (Table 1).

**Table 1:** Ongoing and planned demonstration PrEP (oral and topical) and open label extension projects involving women, men, MSM, sex worker and discordant couples

Study/project	Population	Description	Location	Status
Partners demonstration project	1000 HIV serodiscordant couples	Open label Daily truvada (TVD) oral as bridge to treatment in infected partner. F/up 24 months	Kenya, Uganda	Enrolling Results 2014/15
CAPRISA 008		Open label 1% TDF vaginal gel BAT 24	South Africa	Enrolling Results 2013
CHAMPS-SA PrEP	150 young men and women (15–19 yr)	Open label TVD oral	South Africa	Enrolling Results 2015/6
SAPPHIRE FSW RCT	2800 FSW	Open label Oral daily TVD	Zimbabwe	Enrolling
TAPs: Expanded use of ART for treatment and prevention for female sex workers in South Africa	400 FSW	Open label PrEP and immediate ART for FSWLWHIV	South Africa	Enrolling
Sibanye MSM Project	200 MSM	Open label (adult MSM)	South Africa	Enrolling Results 2015/6
PrEP for MSM	300 MSM	Open label (adult MSM)	South Africa	Under review
RCTS with planned open label extensions	Population	Description	Location	Status
ASPIRE	2629 hetero women	Placebo RCT Dapivirine vaginal ring	Zimbabwe, Malawi, Uganda, South Africa	Enrolling 2015
RING study	1959 hetero women	Placebo RCT Dapivirine vaginal ring	South Africa	Enrolling 2015

### PrEP in southern/eastern Africa: implementation

There are several opportunities to provide PrEP within the region in a way that is focused and allows for sufficient levels of accountability and safety. In most cases, the feasibility is linked to the availability of established service platforms into which PrEP distribution could be integrated. A number of options are considered as follows (Table 2):

1. Adolescent girls: Girls and young women between 15 and 30 years old have extraordinary high incidence in South Africa [30–32]. The most recent household survey confirms the extreme high risk of the epidemic nationally among adolescent girls, with 15–19 year olds four times and young women in their twenties eight fold more likely to be infected than their male counterparts [32]. While there is no doubt this population could benefit from novel prevention interventions, it is less clear which service platform would be most appropriate. While there has been opposition to schools-based expanded sexual education programmes, accessing young women after they have left school is challenging, and most girls attend school until at least 16 years of age. Providing reproductive health services, including education and PrEP, at schools prior to exiting, would allow young women to be proactive in accessing services on leaving school. That said, while an accessible, captive opportunity, this platform may not be attractive for reasons of labelling and stigmatization [33, 34]. Alternatively, reviving the concept of adolescent-friendly health services and exploiting other community-based venues where adolescents share spaces and already tend to congregate may be another entry point. This may include youth drop-in centres and other testing and screening opportunities. Although not yet well developed country wide, the South African government has voiced concern about the high rate of infections in adolescent girls and may be amenable to innovative out-of-health-facility options. Johnson *et al*. compared PrEP given to five age groups in South Africa: 15–19, 20–24, 25–34, 35–49 and 50 or older. For both males and females, the age group in which it was most efficient to promote PrEP was the 15–19 age group [35], with the efficiency being greater in females than in males in this age group. Individuals who acquire HIV at younger ages have greater future potential to transmit HIV than people at older ages, and so prevention here has the greatest potential public health and cost benefit [35]. While incidence of infection among the general population of young women elsewhere in the region is lower than in South Africa, it remains very high in certain key populations, notably female SWs and MSM [36, 37]. Incidence not withstanding uptake and adherence may differ by age group due to differences in access to health services, provider attitudes about sexuality, self-agency and influence of peers and other factors that may influence adherence to pill taking.
2. Women's contraceptive services: In Southern Africa, there is extensive contraception coverage within clinics, including more recently implants, injectables, condoms and oral medication. Clinics which provide contraception could identify a high-risk population (young, sexually active women) who, if found to be HIV-negative, could be offered PrEP, along with sexual health counselling although this will depend on the setting (in some countries in the region, contraception is most easily accessible to married women after the birth of their first child).
3. Services directed at MSM: In the last decade, there has been increasing surveillance and research in the population of MSM in Africa – as in every part of the world, HIV rates have been found to be higher than the background male population rates in both gay identified and non-identified MSM [38]. There is less information in transgendered populations, but where studied, transgendered women have the highest rates of HIV [39]. Same-sex sexual relations are legal in South Africa and have been since the early 90s. Elsewhere in the region, same-sex relationships are criminalized. Throughout, public sector health services remain very heterocentric and are well known not to encourage or even enable male attendance. In addition, there is increasing recognition that men are a population being missed by HIV testing services and other prevention services [40] in general, and this is compounded in the case of MSM [41]. To this end, there has been increasing efforts to enhance male involvement in health services, scaling up sensitization of health workers to gender diversity (and in some countries) MSM sexual health training. In South Africa, there are an increasing number of clinics that are accredited to have the skill set to look after MSM. In addition, there is a strong network of Southern African GPs who have provided MSM-friendly health care to MSM for many years and may be an established and knowledgeable network to exploit.
  Brookmeyer and colleagues looked at agent-based modelling of the effectiveness of HIV prevention packages for MSM in South Africa and considered packages consisting of four components: ART; PrEP for high-risk uninfected persons; behavioural interventions to reduce rates of unprotected anal intercourse (UAI); and campaigns to increase HIV testing. They found that a four component package consisting of a 15% reduction in the rate of UAI, 50% PrEP coverage, 50% reduction in persons who never test for HIV, and 50% increase in ART coverage could prevent 33.9% of infections over 5 years (95% confidence interval, 31.5–36.3). The package components with the largest incremental prevention effects were UAI reduction and PrEP coverage. The impact of increased HIV testing was magnified in the presence of PrEP [42].
4. Sex workers: South Africa has an expanding verticalized SW programme, partly government and partly donor funded, often combined with other programmes, such as trucker heath programmes. Likewise in Zimbabwe, there is a national network of clinical services specifically for SWs supported by a peer educator delivered community empowerment programme. Kenya has articulated the need for a specific SW prevention programme. The clinical care for SWs is essentially a reproductive health package with intensive HIV testing, and integrating PrEP would be relatively simple. While risk compensation and subsequent “condom migration” is a potential concern, in reality, many women struggle to use condoms consistently [43]. The potential implications for other sexually transmitted infections (STIs) and unintended pregnancy need to be added to the broader health implications of promoting antiretroviral-based prevention methods if this risk is likely [44].
  In South Africa, it has been estimated that between 6 and 11% of adult HIV transmission has been attributable to commercial sex, but in other regions in which the HIV epidemic is more concentrated, SW-specific interventions may be relatively more important [44]. For example, other models suggest that providing a topical gel to SWs would reduce HIV incidence in the general population by only 9% in the South African context, compared to 48% in Benin [45], where commercial sex is estimated to account for more than half of HIV infections in men [46]. Previous modelling has also shown that SW interventions are likely to have relatively less impact in mature epidemics than in early-stage epidemics [47]. Bekker *et al*. recently published a model simulation based on the South African heterosexual epidemic which indicated that condom promotion and distribution programmes in South Africa had already reduced HIV incidence in SWs and their clients by more than 70%. Under optimistic model assumptions, PrEP together with ‘test and treat’ programmes could further reduce HIV incidence in South African SWs and their clients by 40% or more in future. The authors suggested that the addition of these biomedical approaches to a prevention package that included behavioural and structural components in consultation with SW communities would go far in reducing HIV infection in sex work in many different settings worldwide [44].
5. Pregnant women: Most women in South Africa deliver within a health facility; HIV testing, PMTCT and initiation of ART during pregnancy has reached relatively high levels. Some studies have demonstrated high levels of seroconversion, and may account for some of the small percentage of PMTCT failures. Initiating PrEP during pregnancy has some theoretical toxicity concerns for the foetus, but the antenatal verticalized service would be ideal to initiate and maintain HIV-negative pregnant women [48].
6. Serodiscordant couples: Serodiscordant couples make up an appreciable proportion of all partnerships in the region, and UNAIDS estimates this is where the greatest number of transmissions occur [49, 50]. While national and international guidelines have identified serodiscordant couples as a priority for treatment as prevention (where the index partner takes ART regardless of CD4 count), using PrEP during the period before virological suppression of the index partner is achieved or in situations where the index partner declines to start or is poorly adherent to treatment may be indicated. The option of PrEP for serodiscordant couples could be discussed at HIV testing services or within treatment services. Additionally, there are limited options for safe conception for serodiscordant couples in the region, a problem when the desire and cultural expectation for couples to have children is strong [51, 52]. Peripartum PrEP to cover the period of conception and pregnancy has the potential to reduce HIV acquisition in this specific circumstance [53, 54].
  Extending access beyond this, especially to heterosexual men, may require a focus on workplaces and other venue-based contact. Drug users, including injecting drug users, have also been shown to derive benefit from PrEP [55]. The region has a growing population of injecting drug users, and it will be important to consider PrEP in any combination package for this risk group. Targeting also assumes nuanced understanding of where highest incidence is present. This idea of tracking “hot spots,” while apparently an efficient deployment of this prevention tool requires sophisticated, flexible and dynamic surveillance with huge flexibility in health systems to quickly and effectively respond. On the other hand, targeting high-burden areas and/or populations may offer a rational, phased approach to general prevention scale-up [56].

**Table 2:** Potential population types suitable for implementation of pre-exposure prophylaxis prevention strategies in Southern Africa

Population type	Potential venue	Demo. projects underway in the region	Potential barriers
Adolescent girls	School health services, community-based adolescent venues, adolescent-friendly health services	√	Consent, participation, adherence, regular testing
Contraceptive services	Contraception services	×	Participation, adherence, regular testing
MSM	Mens’ health services, MSM specific services	√	Regular testing, available services, homoprejudice and criminalization
Sex workers (SWs)	SW services, SW venues	√	Criminalization, regular testing, labelling and stigma
Pregnant women	Antenatal care services	×	Participation, regular testing, continued use
Discordant couples	Safer conception services, HIV care services	√	Participation, adherence, regular testing
Men	Work venues	×	Acceptance by work health programmes, regular testing

### PrEP in southern/eastern Africa: ethical issues

The ethics of PrEP have been extensively discussed in the HIV and ethics literature [57, 58]. In summary, concerns have largely centred on displacement of antiretroviral resources for treatment, prevention in HIV-negative people, issues of resistance, and sexual risk disinhibition. We believe that PrEP raises no new PrEP-specific ethical issues and that dilemmas may be tackled using conventional ethical frameworks, for all the ethical concerns raised within the literature.

Authors have argued on the displacement issue that this is a conventional debate around resource allocation and that balancing prevention and treatment is relatively straightforward; treatment takes precedence when it comes to allocation of antiretroviral products, although even this stance has been challenged [59]. In South Africa, there is currently adequate budget for treatment, and extensive resources allocated to prevention, although there are substantial funding gaps elsewhere in the region [60]. The tenofovir/emtricitabine combination is available at a fraction of the cost of what it is sold for in developed countries. Commercial cost of generic tenofovir is currently around $10 per month, and generic tenofovir/emtricitabine is approximately $30 per month. Government tenders drive these prices even lower. So, rationing due to cost may be less of an issue than it would be in poorer nations, and paradoxically due to low cost could be more available than in richer countries.

Resistance has not been a problem in the clinical trials to date; empiric research will certainly be tracking this, but this is more of a possible future public health threat than a current ethical dilemma. New classes of drugs, as well as drugs with higher resistance barriers within existing classes, have been licensed at extraordinary low cost within the country, meaning that alternatives are likely, even if worst case scenarios around drug resistance occur. However, modelling at least suggests that PrEP is unlikely to pose a problem for antiretroviral resistance [36]. Interestingly, a number of published model analyses have suggested that HIV drug resistance in a population would be largely driven by ART, not PrEP, likely as a result of insufficient ART adherence or lack of viral load monitoring in ART programmes, leading to selection of resistant variants during incomplete viral suppression [36].

The issue of disinhibition or greater risk taking in the face of perceived added protection, has been much debated for all types of HIV prevention, including medical male circumcision, condoms and microbicides, and follows even older debates around contraception and the ‘morning-after’ pill. While causing much theoretical concern, very little tangible evidence exists for this phenomenon. Yet this remains a source of uncertainty when considering the potential effect of oral and topical PrEP. Some modelling studies have also raised this concern [20, 61, 62].

### PrEP in Southern Africa: financing

South Africa is a middle-income country, with a well-resourced private sector and an extensive state funded programme for HIV/AIDS treatment and prevention. The state HIV treatment programme is almost totally financed from the national fiscus, with some key donor support [1]. Prevention programmes including PrEP provision within these programmes are possible but must be sourced. The South African national treasury has used similar modelling to plan for the budgeting of the broad antiretroviral programme and would probably look favourably to proposals addressing this provision. Other countries in the region are largely considered low-income countries, with a much higher proportion of HIV prevention and care funded through donors [60]. However, the case for investing in PrEP, particularly for those at highest risk, remains sound. Kenya in particular has written PrEP into its plan for HIV eradication by 2030 [63].

The price of currently available PrEP is a fraction of that in developed countries, with generic competition. There is accumulating evidence that tenofovir alone is adequate for protection and, if confirmed, may mean the price of PrEP falls further.

## Conclusions

In sub-Saharan Africa there were an estimated 1.6 million new HIV infections in 2012 alone [64]. With a regional health system that is buckling under the load of HIV and TB diagnosis, linkage, care and retention, finding effective and easily implementable ways to reduce new HIV infections is an important undertaking. Targeted PrEP and tailored combination prevention including PrEP may well provide a useful additional intervention in Africa's ongoing movement towards epidemic control.
